# A knowledge-driven deep learning framework for organoid morphological segmentation and characterization

**DOI:** 10.1186/s12915-025-02411-8

**Published:** 2025-10-21

**Authors:** Yiming Qin, Jiajia Li, Yin Heng, Zheyuan Wang, Dezhi Wu, Mahi Rahman, Pengwei Hu, Tobias Plötz, Alexander Hopp, Nicholas Kurniawan, Mathias Winkel, Philipp Harbach, Chunling Tang, Feng Tan

**Affiliations:** 1 School of Clinical Medicine, Tsinghua University, Beijing, China; 2 Merck KGaA, Darmstadt, Germany; 3 School of Chemistry and Chemical Engineering, Shanghai Jiao Tong University, Shanghai, China; 4 Department of Computer Science and Engineering, Shanghai Jiao Tong University, Shanghai, China; 5 The Xinjiang Technical Institute of Physics and Chemistry, Chinese Academy of Science, Urumqi, China; 6 Institute for Complex Molecular Systems, Eindhoven University of Technology, Eindhoven, Netherlands; 7 Centre for Craniofacial & Regenerative Biology, King’s College London, London, UK

**Keywords:** Organoid, Deep learning, Knowledge-driven

## Abstract

**Background:**

Organoids have great potential to revolutionize various aspects of biomedical research and healthcare. Researchers typically use the fluorescence-based approach to analyse their dynamics, which requires specialized equipment and may interfere with their growth. Therefore, it is an open challenge to develop a general framework to analyse organoid dynamics under non-invasive and low-resource settings.

**Results:**

In this paper, we present a knowledge-driven deep learning system named TransOrga-plus to automatically analyse organoid dynamics in a non-invasive manner. Given a bright-field microscopic image, TransOrga-plus detects organoids through a multi-modal transformer-based segmentation module. To provide customized and robust organoid analysis, a biological knowledge-driven branch is embedded into the segmentation module which integrates biological knowledge, e.g. the morphological characteristics of organoids, into the analysis process. Then, based on the detection results, a lightweight multi-object tracking module based on the decoupling of visual and identity features is introduced to track organoids over time. Finally, TransOrga-plus outputs the dynamics analysis to assist biologists for further research. To train and validate our framework, we curate a large-scale organoid dataset encompassing diverse tissue types and various microscopic imaging settings. Extensive experimental results demonstrate that our method outperforms all baselines in organoid analysis. The results show that TransOrga-plus provides comparable analytical results to biologists and significantly accelerates organoid work process.

**Conclusions:**

In conclusion, TransOrga-plus integrates the biological expertise with cutting-edge deep learning-based model and enables the non-invasive analysis of various organoids from complex, low-resource, and time-lapse situations.

**Supplementary Information:**

The online version contains supplementary material available at 10.1186/s12915-025-02411-8.

## Background

Organoids are three-dimensional structures that mimic the architecture and function of organs in a miniaturized and simplified form [1]. They are derived from stem cells or tissue samples and cultured in vitro, providing a more physiologically relevant model compared to traditional two-dimensional cell cultures. In biomedical [2–7] and healthcare research [8–11], organoids are beginning to demonstrate their revolutionary potential. For example, they can be used to model human diseases more accurately than traditional cell cultures, allowing researchers to better understand disease mechanisms and identify potential treatments.

To cultivate organoids, researchers are investigating a bio-engineering approach for fast and stable in vitro culturing. One key aspect of bio-engineering organoids is to be able to longitudinally analyse their growth dynamics, such as the organoid cells morphology, population distribution, and time-course variance. However, researchers and practitioners still face several challenges when performing their analytical work. First, organoids grow in complex culture media with interference factors, such as air bubbles and nutritional debris. These interference factors are not static and change over time. Second, organoid dynamics is an integrated process characterized by morphological heterogeneity. Different types of organoids exhibit diverse morphologies, and even within the same type, their morphology can vary over time. Additionally, the occurrence of organoid cell connections and overlapping during growth further complicates the analysis of organoid dynamics.

Existing methods typically use fluorescence staining microscopic images or genetically modification [11–15] to conduct organoid dynamics analysis. Researchers first stain the organoids with specific fluorescent dyes that help differentiate organoid cells from the culturing medium. They then label the organoids, either manually or with computer assistance, and empirically set microscopic parameters to conduct growth analysis. However, these methods are invasive and highly resource intensive. Some fluorescence dye-based approaches may disrupt the intrinsic cellular dynamics of the original samples [16, 17] or induce cumulative toxicity due to prolonged culture periods and restricted diffusion within the hydrogel matrix [18]. Moreover, these methods require the additional purchase of fluorescence dyes and professional dyeing, resulting in high resource overhead. Therefore, there is a growing need for an automatic, non-invasive, and low-resource approach [19–23]. Previous researchers proposed non-invasive and low-resource approaches using bright-field or phase-contrast microscopic images [24–29]. Furthermore, specialized imaging platforms have been developed for label-free, non-invasive assessment of cellular viability [30–33]. However, these methods commonly suffer from limited robustness and generalizability. First, compared to the fluorescent images, the bright-field or phase-contrast microscopic images lack colour and texture context, which poses a challenge to the robustness of the image analysis. Second, current deep learning-based methods heavily depend on many organoid samples for training and lack of domain knowledge, resulting in poor generalizability. However, in real-world practice, organoid samples are limited, and the biologists’ approach is not solely based on images. Instead, they consider both the images and biological knowledge, such as the tissue type and culture medium elements, to make analytical decisions. Additionally, the design of existing methods still has inherent flaws in organoid dynamics analysis. For instance, the pyramid structure in the feature extraction of classical deep learning-based recognition methods can result in unavoidable information loss and consequently inaccurate organoid detection [28].

To address these gaps, we propose a knowledge-driven deep learning framework, named TransOrga-plus, for organoid dynamics analysis. Given the bright-field microscopic image and the biological knowledge provided by scientists, TransOrga-plus automatically detects, tracks, and analyses cellular dynamics. Our model mainly contains three modules, a biological knowledge-driven branch embedded multi-modal segmentation module, a tracking module and an analysis module. Unlike traditional hand-crafted formulas, the term biological knowledge in our work specifically refers to image-based morphological characteristics of organoid-derived cells as recognized by domain experts—such as shape, size, texture, edge contrast, and compactness. These features, grounded in biological expertise, are used to differentiate meaningful organoid-derived cells from bright-field microscopy images. The biological knowledge-driven branch extracts the features from the biological knowledge provided by the user, and then fuses the extracted features with the whole image features to guide the analysis. To accurately detect organoids, we develop a multi-modal transformer-based model that utilizes the frequency domain features to provide morphological clues and the spatial domain features to provide visual clues. The tracking module decouples identity features and visual features of organoids for lightweight multi-organoid tracking. Finally, the analysis module outputs the single-organoid analysis, bulk analysis, time-course analysis. Extensive experimental results demonstrate that TransOrga-plus outperforms all baselines in organoid dynamics analysis and achieves comparable performance to manual ways. Additionally, TransOrga-plus successfully completes customized organoid dynamics analysis based on feedback from biologists.

Specifically, our novelties and contributions can be summarized as follows:

1. We proposed TransOrga-plus, a knowledge-driven deep learning framework for robust and customizable organoid dynamics analysis. The framework integrates detection, tracking, and human-in-the-loop feedback to adapt to diverse experimental conditions and morphological variability.
2. We designed a biological knowledge-driven module that incorporates domain-specific prior knowledge, interactively or explicitly, into the learning process. This module guides detection and tracking with interpretable biological constraints and improves generalizability with limited data by reducing reliance on extensive annotations.
3. To train and validate our learning framework, we curated a large-scale dataset from both internal and external scenarios. This dataset covers a wide range of tissue types at different maturity phases. Extensive experimental results show that our framework not only outperforms all baselines in detection and tracking accuracy but also generalizes to various tissue types.

## Results

### Overall framework

**Fig. 1:**
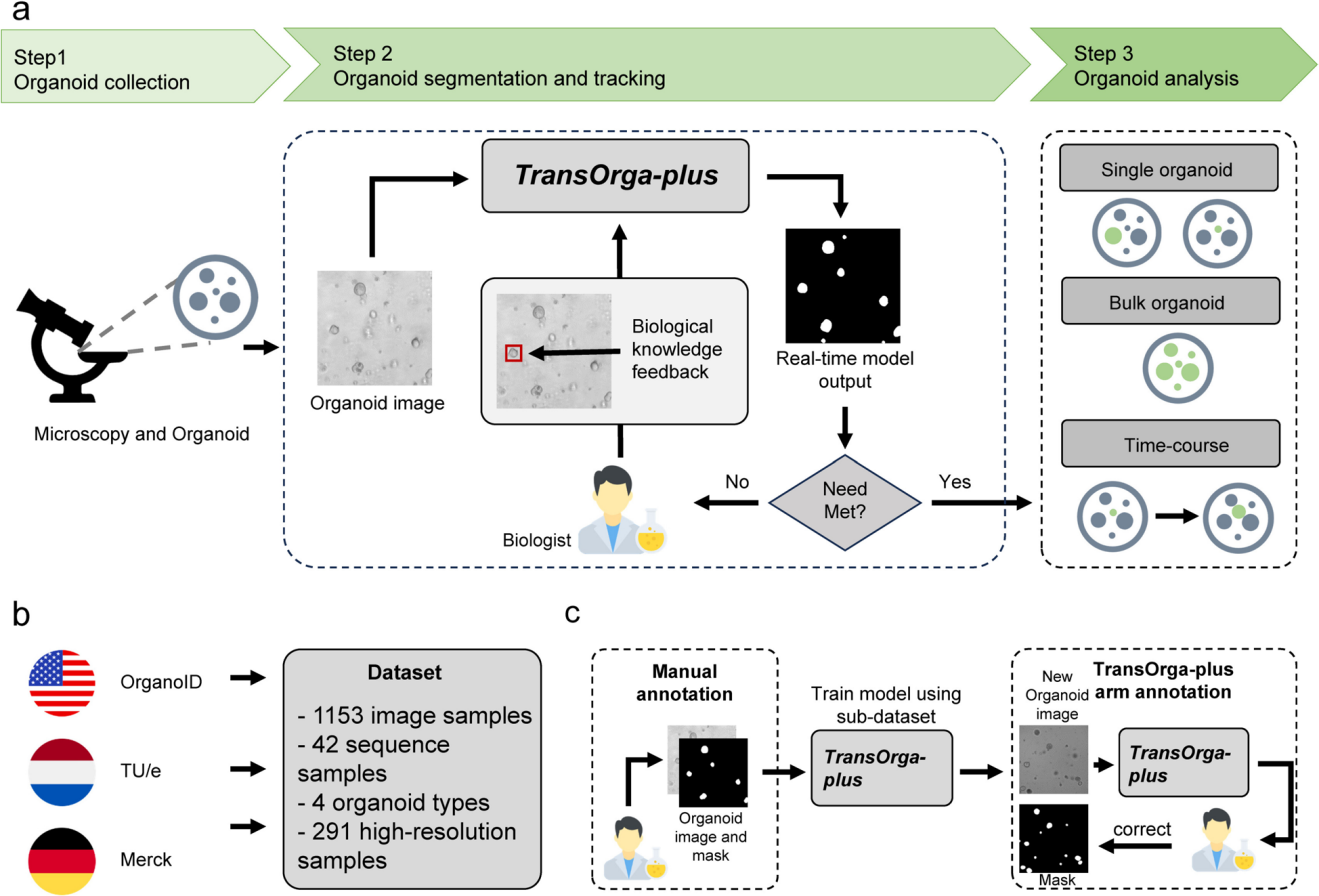
The overview of TransOrga-plus and our dataset. **a** The overview of TransOrga-plus. Given bright-field microscopic images and the biological knowledge provided by scientists, TransOrga-plus automatically and interactively detects, tracks, and analyses organoids. **b** Dataset. Our dataset includes OrganoID, TU/e, and Merck. **c** Dataset annotation. The hybrid way is designed to annotate our dataset efficiently. Biologists first manually select and annotate a sub-dataset. TransOrga-plus is trained on the sub-dataset. Using the trained TransOrga-plus, biologists obtain coarse masks of left samples and then correct them to produce final annotations

The overview of TransOrga-plus is shown in Fig. 1a. Given the bright-field microscopic image and biological knowledge, TransOrga-plus automatically analyses organoids based on biological knowledge. TransOrga-plus mainly contains three modules: a biological knowledge-driven branch embedded multi-modal segmentation module, a light-weight tracking module and an analysis module. The multi-modal segmentation module utilizes visual and frequency domain clues to detect organoids from bright-field microscopic images. Using the biological knowledge-driven branch, we integrate user-provided biological knowledge into the model and guide it to generate personalized analysis results. The lightweight tracking module is designed to suit the high-throughput organoids. The analysis module utilizes the detection and tracking results to complete organoid dynamics analysis. To train and validate our model, we build a large-scale dataset that contains different organoid types. The collection of our dataset is shown in Fig. 1b and c.

### Large-scale organoid dataset

To train and evaluate the proposed framework, we curated a large-scale dataset by following these procedures, as shown in Fig. 1b. The raw data came from the OrganoID [28], Eindhoven University of Technology (TU/e) [34], and Merck (Table 1). Our large-scale dataset comprises 1153 bright-field microscopic images, encompassing a diverse array of organoid types and various imaging settings. The organoid types include salivary adenoid cystic carcinoma (ACC), colon epithelia (Colon), lung epithelia (Lung), pancreatic ductal adenocarcinoma (PDAC), and mammary (Mammary). Regarding image settings, we have 862 images with a resolution of 512 × 512 pixels and 291 high-resolution images (1024 × 1024 pixels or higher). Additionally, our dataset features 42 microscopic image sequences, each capturing a 92-h growth process of organoids. For data labelling, we adopted a hybrid annotation approach which contains manual annotation and annotation with TransOrga-plus assistance (Fig. 1c). The detailed data curation process is described in the “Methods” section.

**Table 1:** The curated dataset. Our dataset includes samples from OrganoID, TU/e, and Merck. It contains diverse types of organoids and image settings

Source	Total sample	High-resolution sample	Sequence sample	Tissue type
OrganoID	66	0	42	Colon, Lung, PDAC, ACC
TU/e	1074	278	0	Lung, Mammary
Merck	13	13	0	Lung

### Accurate, robust, and generalizable organoid detection

We conduct comparative experiments using our dataset to investigate TransOrga-plus’s capability to accurately, robustly, and generally detect complex organoids in microscopic images. We benchmark TransOrga-plus with the following state-of-the-art methods: SegNet [35], A-Unet [36], StartDist [37], CellPose [38], ilastik [18], and OrganoID [28]. The training and validation datasets contain different tissue types of organoids, including ACC, Colon, Lung, and PDAC.

**Fig. 2:**
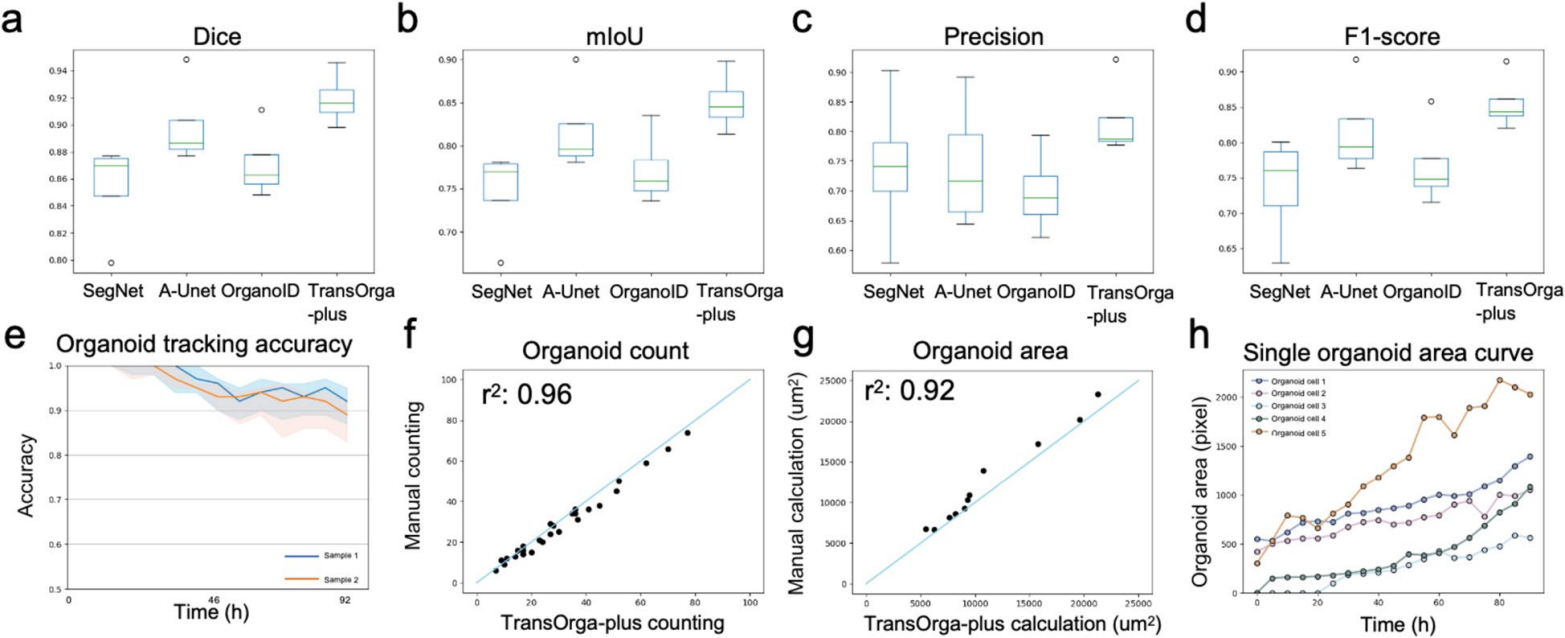
Results of organoid morphological segmentation and characterization. **a** The Dice score of SOTA methods and our method. **b** The mIoU score of SOTA methods and our method. **c** The Precision score of SOTA methods and our method. **d** The F1-Score score of SOTA methods and our method. The analysis used a paired Student *t* test. **e** Tracking accuracy. We calculate the tracking accuracy of TransOrga-plus based on the manual tracking. **f** Organoid count. The *x*-axis indicates the number counted by biologists and the *y*-axis indicates the number counted by our method. **g** Organoid area calculation. The *x*-axis indicates the area calculated by biologists, and the *y*-axis indicates the area calculated by our method. **h** Single organoid area curve. We recorded five organoid cells area change curves over time using TransOrga-plus

**Fig. 3:**
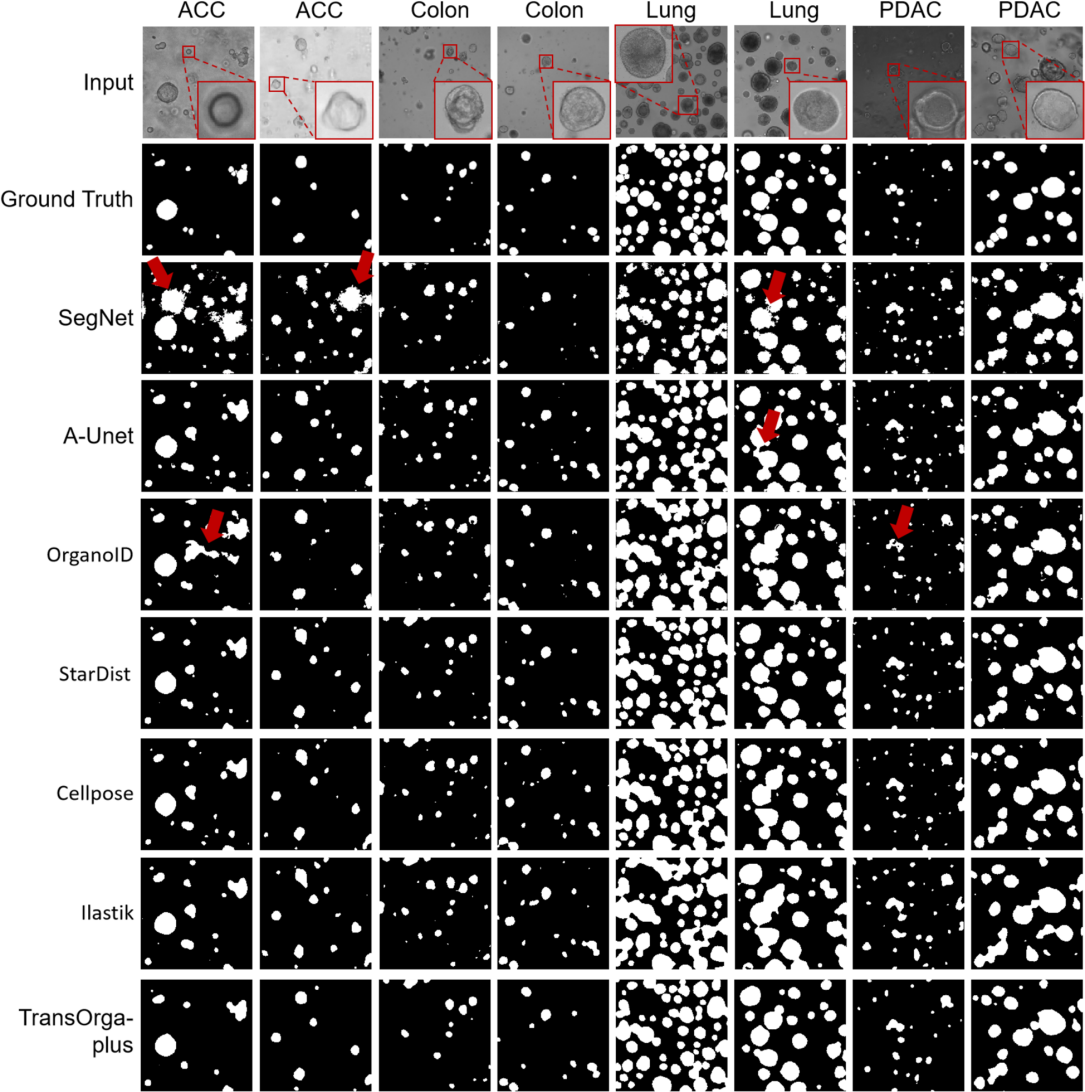
Comparison results of organoid detection. We compared our method with baselines on different types of organoids. Our method achieves the best results. We highlight the failure cases using red arrows

The Dice measures the overlap between the predicted segmentation mask and the ground truth mask. Mean Intersection over Union (mIoU) is a popular metric for evaluating the performance across negative (background) and positive (organoid) classes in segmentation tasks, with 1 being perfect segmentation. Precision measures the proportion of correctly predicted positive (organoid) pixels out of all predicted positive pixels. Recall measures the proportion of correctly predicted positive pixels out of all actual positive pixels. The F1 Score is the harmonic mean of Precision and Recall, balancing the two. It is especially useful when a balance between Precision and Recall is desired. A high F1-score indicates a model that has both high recall and precision, which is essential when both false positives and false negatives are critical in segmentation accuracy. As shown in Table 2 and Fig. 2a–d, compared with baselines, TransOrga-plus demonstrates excellent quantitative performance with Dice 0.919 $\pm$ 0.02, mIoU 0.851 $\pm$ 0.04, precision 0.819 $\pm$ 0.07, recall 0.904 $\pm$ 0.01 and F1-score 0.856 $\pm$ 0.04 (all *P* < 0.001). The qualitative results are shown in Fig. 3. The baseline methods produce false and broken detections, as indicated by red arrows, due to medium bubble and debris interference, whereas our approach is resistant to such interference. Additionally, baseline methods lack constraints on generating segmentation with rational shapes, resulting in irregular and fragmented outputs, as indicated by red arrows.

**Table 2:** Quantitative results of baselines and our method on different tissues. We compute the Dice, mIoU, Precision, Recall, and F1-score of different methods on various types of organoids. The upward arrow indicates that a higher score is better. We highlight the best results using bold

Model	Dice $\uparrow$	mIoU $\uparrow$	Precision $\uparrow$	Recall $\uparrow$	F1-score $\uparrow$
SegNet [35]	$0.853\pm 0.04$	$0.746\pm 0.06$	$0.741\pm 0.13$	$0.789\pm 0.05$	$0.738\pm 0.08$
A-Unet [36]	$0.899\pm 0.03$	$0.818\pm 0.06$	$0.743\pm 0.11$	$\mathbf0\boldsymbol.\mathbf{931}\boldsymbol\pm\mathbf0\boldsymbol.\mathbf{04}$	$0.818\pm 0.07$
OrganoID [28]	$0.871\pm 0.03$	$0.773\pm 0.04$	$0.698\pm 0.07$	$0.872\pm 0.05$	$0.768\pm 0.06$
StartDist [37]	$0.791\pm 0.01$	$0.654\pm 0.01$	$0.721\pm 0.02$	$0.876\pm 0.01$	$0.791\pm 0.02$
CellPose [38]	$0.754\pm 0.01$	$0.605\pm 0.01$	$0.642\pm 0.02$	$0.913\pm 0.01$	$0.754\pm 0.03$
Ilastik [18]	$0.767\pm 0.01$	$0.622\pm 0.02$	$0.728\pm 0.01$	$0.810\pm 0.01$	$0.767\pm 0.01$
Ours	$\mathbf0\boldsymbol.\mathbf{919}\boldsymbol\pm\mathbf0\boldsymbol.\mathbf{02}$	$\mathbf0\boldsymbol.\mathbf{851}\boldsymbol\pm\mathbf0\boldsymbol.\mathbf{04}$	$\mathbf0\boldsymbol.\mathbf{819}\boldsymbol\pm\mathbf0\boldsymbol.\mathbf{07}$	$0.904\pm 0.01$	$\mathbf0\boldsymbol.\mathbf{856}\boldsymbol\pm\mathbf0\boldsymbol.\mathbf{04}$

Regarding scalability to high-resolution images, the model performances are evaluated on high-resolution microscopic images (1024 × 1024 or higher) where models’ structure scalability can be assessed as well. Additionalfile 1: Fig. S1 shows that the state-of-the-art (SOTA) OrganoID suffers from performance degradation. However, TransOrga-plus maintains robust detection performance for high-resolution images.

To evaluate the effectiveness of our proposed modules, we also conducted an ablation study. In the ablation experiments, we remove the proposed modules one by one and retrain the model lacking the module to get the results. The ablation study results shown in Table 3 indicate that all proposed modules positively impact organoid recognition.

**Table 3:** Quantitative Results of Ablation Study. Our proposed components contribute significantly to model performance. The upward arrow indicates that a higher score is better. We highlight the best results using bold

Model	Dice $\uparrow$	mIoU $\uparrow$	Precision $\uparrow$	Recall $\uparrow$	F1-score $\uparrow$
Ours without multi-modal	$0.879\pm 0.03$	$0.789\pm 0.06$	$0.692\pm 0.04$	$0.916\pm 0.02$	$0.776\pm 0.03$
Ours without ${\mathcal{L}}_{\text{com}}$	$0.841\pm 0.03$	$0.711\pm 0.02$	$0.579\pm 0.10$	$\mathbf0\boldsymbol.\mathbf{930}\boldsymbol\pm\mathbf0\boldsymbol.\mathbf{02}$	$0.719\pm 0.06$
Ours without biological knowledge	$0.882\pm 0.03$	$0.823\pm 0.03$	$0.726\pm 0.06$	$0.890\pm 0.01$	$0.800\pm 0.03$
Ours	$\mathbf0\boldsymbol.\mathbf{919}\boldsymbol\pm\mathbf0\boldsymbol.\mathbf{02}$	$\mathbf0\boldsymbol.\mathbf{851}\boldsymbol\pm\mathbf0\boldsymbol.\mathbf{04}$	$\mathbf0\boldsymbol.\mathbf{819}\boldsymbol\pm\mathbf0\boldsymbol.\mathbf{07}$	$0.904\pm 0.01$	$\mathbf0\boldsymbol.\mathbf{856}\boldsymbol\pm\mathbf0\boldsymbol.\mathbf{04}$

We further conducted an organoid type classification task across four categories: ACC, Colon, Lung, and PDAC. Our model demonstrated robust performance, achieving an average classification accuracy of 92.86% ± 2.28%, indicating its strong ability to distinguish between different organoid types based on morphological or structural features.

### Consistent tracking of organoid growth and cellular viability analysis

We conduct tracking experiments to evaluate TransOrga-plus’s ability to track organoid growth consistently. The microscopy image sequences were formed by taking snapshots of the organoids in the medium every 2 h over a period of 92 h. TransOrga-plus takes the microscopy image sequence as input and produces numerically labelled tracking results. Specialized biologists performed organoid tracking task on the same microscopy image sequence to serve as ground-truth labels. Tracking accuracy is defined as the fraction of identified organoids that are correctly matched at each time step [28]. We also performed tracking experiments at different time intervals, e.g. 4, 6, 12 h, by discarding intermediate frames.

We compared our method with original segment-and-track anything (STA) [40, 40] and fine-tuned STA using our dataset. The results, as shown in Additional file 1: Fig. S2, indicate that our method successfully captures the growth trajectory of organoids at different time steps, demonstrating TransOrga-plus’s advantage of integrating biological knowledge for long-term tracking. The original segment anything model (SAM) shows poor generalization on organoids. The results of the tracking accuracy are shown in Fig. 2e, which range from 92.1 to 94.4%. The temporal consistency in tracking results allows for the observation of dynamic phenotypic changes at the single-cell level, which have been associated with cellular viability [30]. A video demonstration of the tracking capabilities is also available on our official website (https://github.com/dev-csftan/TransOrga).

### Performance improved by biological knowledge

To assess the benefits of the knowledge-driven module in TransOrga-plus, we conducted interactive experiments with emulated biologist feedback. In the interactive experiments, given a microscopy image, (1) TransOrga-plus outputs the initial detection result; (2) based on the initial detection result, the biologist provides specific knowledge, i.e. organoid morphology, through a bounding box; (3) conditioned on the extracted biological knowledge, TransOrga-plus outputs the refined detection result.

**Fig. 4:**
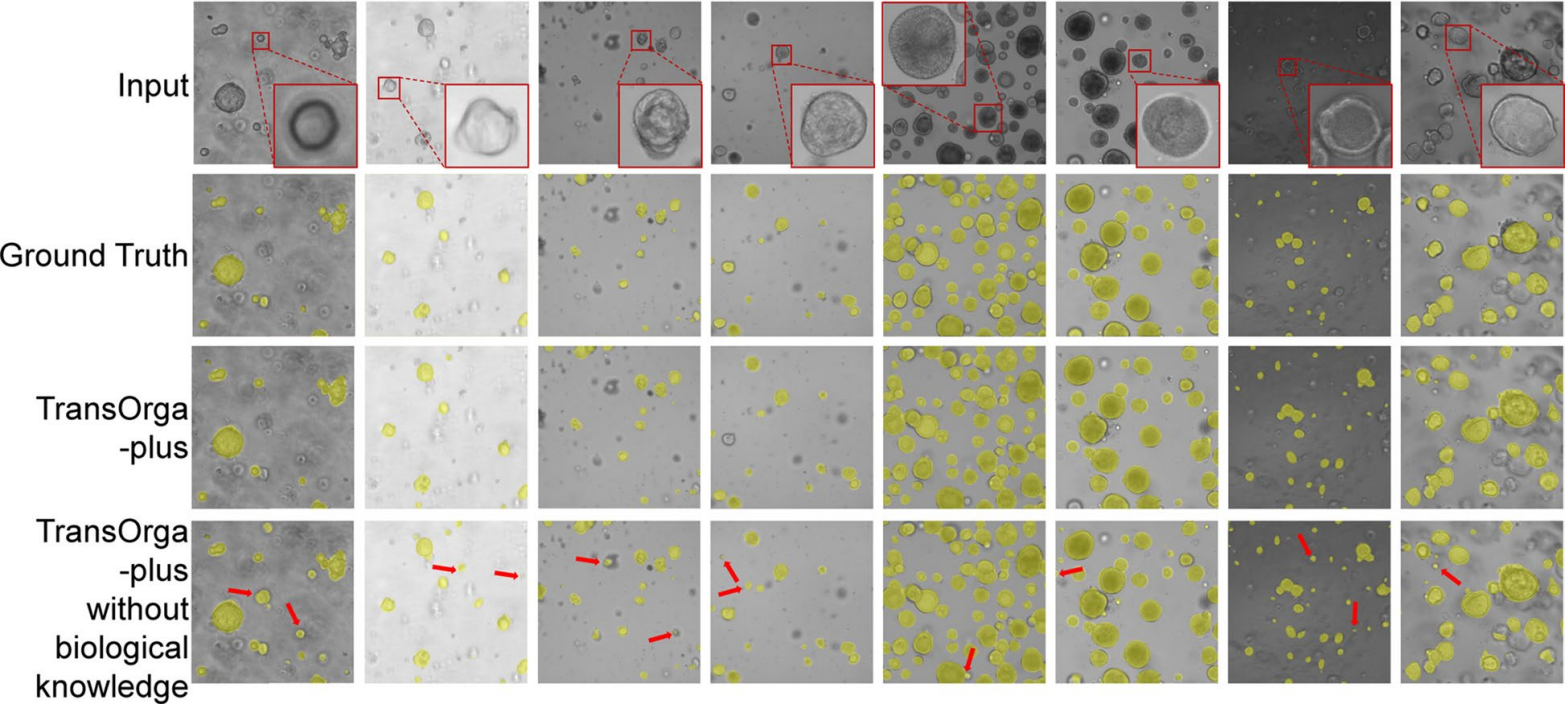
Comparison results of organoid detection conditioned on biological knowledge (i.e. organoid morphology). We compared the results of our method with and without integrating biological knowledge. The yellow region represents the detection region. Error regions are highlighted with red arrows. The results indicate that biological information has a positive impact on detection outcomes

We validated the knowledge-driven process on various tissue types with different biological insights. We also utilized the detection metrics for quantitative analysis. As shown in Fig. 4, the biologist selects the organoid morphology in the red box as biological knowledge feedback for the TransOrga-plus. The model refines the detection results for organoid cells with similar morphologies and eliminates those with significant differences, as indicated by the red arrows. The results demonstrate that the knowledge-driven module can handle a diverse range of organoids under complex and incomplete situations. Quantitative metrics, as shown in Table 3, also show that fusing biological knowledge improves the detection performance.

### Automatic and accurate organoid analysis

TransOrga-plus can automatically analyse multiple organoid indicators, including single-organoid analysis, bulk analysis, and time-course analysis. For single-organoid analysis, our method can accurately categorize different types of organoids (Fig. 3). For bulk analysis, we measure the organoid count and areas. We compare the areas calculated using our method with those calculated manually. Due to different scales from various data sources, we counted the number of pixels occupied. The results are shown in Fig. 2 f and g, where each black dot represents a sample. The *x*-axis indicates the results calculated by biologists, and the *y*-axis indicates the results calculated by our method. ${r}^{2}$ is 0.96 for organoid count and 0.92 for organoid area, which indicates that our method achieved results comparable to the manual way. For time-course analysis, we record the single organoid area curve along time. The results are shown in Fig. 2h, which indicate that our method can efficiently track the area changes of different organoid cells.

## Discussion

Organoid dynamics analysis holds significant importance in biomedical and healthcare research. Organoid data is high-throughput data containing a large number of organoid cells. Therefore, there is a critical need for an automatic organoid analysis approach. OrganoID previously introduced a deep learning method to extract organoid information from bright-field microscopic images of organoids. Nonetheless, organoid analysis remains challenging due to the intricate nature of organoids and the scarcity of adequate training samples. In response, we propose TransOrga-plus, a novel image-based and knowledge-driven system for organoid analysis. TransOrga-plus comprises four main modules: a knowledge-fusion-and-guidance module, a multi-modal segmentation module, a lightweight tracking module, and an analysis module. Our model is trained and validated on our large-scale organoid dataset encompassing diverse organoid types and varied image settings. To our knowledge, TransOrga-plus represents the pioneering application of a biological knowledge-driven deep learning approach to organoid analysis and provides an automatic, non-invasive, resource-efficient, and personalized organoid dynamics analysis tool.

Previous studies [24–28] have demonstrated the potential of deep learning methods in detecting organoids from bright-field images. However, these approaches suffer from model structure and are heavily reliant on the size of the training dataset, leading to limited accuracy and poor generalization. In our study, we introduce a transformer architecture augmented with biological knowledge. The transformer effectively captures robust long-range visual features, preserving global context and feature integrity. Moreover, our method excels in handling ultra-high-resolution images, significantly expanding its applicability. Incorporating biological knowledge embeds specific biological characteristics into the model, mitigating reliance solely on dataset knowledge and thereby enhancing generalization.

Organoid dynamics analysis is crucial for advancing biomedical and healthcare research, including elucidating disease mechanisms and developing treatment strategies. Traditional approaches involve staining organoid with fluorescent dyes or built from genetically modified fluorescent organoid cells for detection, tracking, and analysis. However, fluorescence dye-based approaches may disrupt the intrinsic cellular dynamics of the original samples [16, 17] or induce cumulative toxicity due to prolonged culture periods and restricted diffusion within the hydrogel matrix [18].There is a pressing need for non-invasive methods that utilize bright-field imaging. However, compared to stained images, bright-field images often lack sufficient detail. In our study, we propose a multi-modal transformer-based segmentation module designed to detect organoids from bright-field microscopic images. This innovative model integrates frequency domain information to capture morphological cues and spatial domain information to extract visual cues.

Cost-effectiveness plays a pivotal role in organoid research. Biomedical and healthcare studies often require iterative experimentation for achieving desired outcomes, underscoring the necessity of economical approaches. In organoid dynamics analysis, methods utilizing fluorescent dyes not only involve the expense of these dyes but also necessitate specialized staining expertise, significantly elevating research costs. Our approach utilizes bright-field images without requiring staining, thereby automating organoid dynamics analysis and markedly enhancing cost-effectiveness. Moreover, biologists can easily tailor TransOrga-plus using the proposed knowledge-fusion-and-driven module by incorporating biological insights, facilitating personalized organoid dynamic analysis. This capability addresses specific experimental requirements more effectively than previous deep learning-based methods.

The dataset is a crucial limiting factor for organoid dynamic analysis research based on deep learning. In our study, we have curated a comprehensive dataset encompassing various types of organoid tissues and diverse image settings. Our dataset incorporates publicly available OrganoID data, along with proprietary data from TU/e and Merck. By providing this extensive dataset, we establish a robust foundation for advancing deep learning methods in organoid research, paving the way for future innovations in the field.

Our study has Limitations. First, while in our current version, the biological knowledge is primarily provided through visual data, further investigation into integrating other forms such as mathematical formulations and natural language descriptions is warranted. Second, our current model exhibits reduced accuracy in detecting very small organoid cells, necessitating future modifications to the model architecture. Third, our dataset does not encompass all types of organoids. Fourth, our dataset does not contain intracellular structures, the ground truth for viability of the organoids and the 3D structure of organoids, prompting ongoing efforts to expand and diversify our dataset. Besides, the ablation study shows that removing ${\mathcal{L}}_{\text{com}}$ slightly increases detection recall, likely because ${\mathcal{L}}_{\text{com}}$ enforces a shape prior that favours compact object structures, which may cause non-compact organoids to be partially missed or inadequately segmented, thereby lowering recall. In future work, we plan to explore adaptive regularization strategies that modulate compactness constraints based on local context or learned shape distributions. These steps are crucial for enhancing the comprehensiveness and efficacy of our approach in organoid research.

## Conclusions

In conclusion, we have developed TransOrga-plus, a biological knowledge-driven deep learning system comprising a knowledge-fusion-and-guidance module, a multi-modal segmentation module, a lightweight tracking module, and an analysis module. TransOrga-plus represents a non-invasive, cost-effective, and personalized tool for analysing organoid dynamics. This system significantly enhances the efficiency of organoid dynamic analysis, thereby advancing research in organoid-related fields. Extensive experiments demonstrate that TransOrga-plus holds promise as a transformative solution for organoid culturing and research.

## Methods

### Data collection

#### Data sources

We collected our dataset from different sources, including OrganoID, TU/e, and Merck Corporation. OrganoID is a public dataset containing 66 organoid samples. Researchers at TU/e collected 1352 organoid samples. Researchers at Merck collected 13 organoid samples.

#### Data types

All organoid samples were recorded with RGB format microscopic images. The general resolution is $512\times 512\hspace{0.25em}{\text{pixels}}$. We also collected high-resolution microscope images, all of which have resolutions exceeding $1024\times 1024\hspace{0.25em}{\text{pixels}}$. The ground-truth mask is the binary image with the same resolution as its associated microscopic image, where 1 represents the organoid and 0 represents the background.

#### Data labelling

Data labelling is very time-consuming and labour-intensive due to the complex and high-throughput organoids in each microscopic image. To mitigate this issue, we adopted a hybrid labelling method. Firstly, we manually labelled 44 samples of various types of organoids as sub-dataset. Second, we utilized the sub-dataset to train TransOrga-plus and employed various data augmentation techniques, e.g. crop, rotation, and contrast adjustment, to enhance generalization. Third, we utilized the trained TransOrga-plus to infer the coarse masks for samples without the ground-truth masks. Finally, biologists corrected the coarse masks to formulate the ground-truth mask.

### Segmentation module

#### Input preprocessing

To address memory and computational constraints while preserving fine-grained morphological detail for high-density input and high-resolution input, we employed a sliding window (divide-and-conquer strategy) method. The original input image is tiled into overlapping 512 × 512 patches. Each patch is processed independently by the segmentation and tracking modules. The outputs are then stitched back together, using overlap-aware merging and confidence-based voting to ensure spatial coherence and avoid boundary artifacts. This strategy allows the model to maintain high segmentation fidelity while being computationally feasible on standard GPUs. Our current model is unable to reliably detect or segment organoid-derived cells that are smaller than the size of a single visual pixel in the input bright-field microscopy images. This limitation is primarily due to the resolution constraints of the imaging setup and the receptive field of the neural network.

#### Multi-modal encoder

**Fig. 5:**
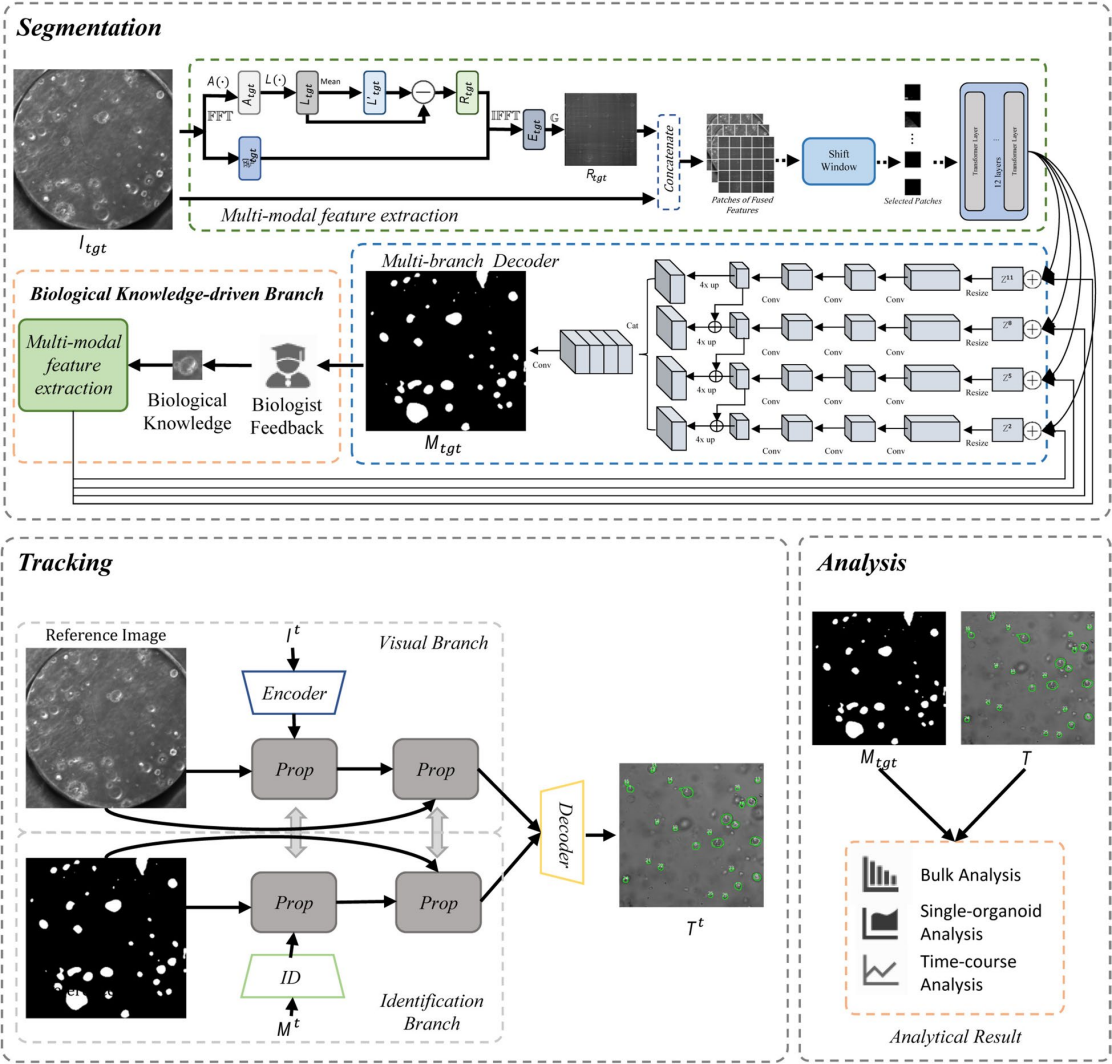
The architecture of TransOrga-plus. TransOrga-plus mainly contains three modules: a biological knowledge-driven branch embedded multi-modal segmentation module, a tracking module and an analysis module. Given the bright-field microscopic image or sequence, the multi-modal segmentation modal recognizes the organoid of each image which is represented as the organoid mask. The biological knowledge-driven branch introduces the biological knowledge feedback from biologists into the model to obtain customized results. Using the sequence of organoid images and segmentation results, the tracking module consistently tracks organoids over time. Based on the segmentation and tracking results, single-organoid analysis, bulk analysis, and time-course analysis are conducted

On the one hand, negative factors in medium and imaging equipment often introduce extra noise into microscopic images. On the other hand, microscopic images lack sufficient colour and texture context for organoids. To solve these challenges, we introduce features of the frequency domain to augment the features in the temporal domain, as shown in Fig. 5. By employing the Fourier transform, we can obtain features in the frequency domain that not only isolate noise but also extract image variations. Given the microscopic image ${I}_{tgt}$, we obtain amplitude ${A}_{tgt}$ and phase spectrum ${P}_{tgt}$ as follows,

1 $$\begin{array}{c}{A}_{tgt}=A\left({\text{FFT}}\left({I}_{tgt}\right)\right)+{e}^{-10}\\{P}_{tgt}=P\left({\text{FFT}}\left({I}_{tgt}\right)\right), \end{array}$$

Where ${\text{FFT}}\left(*\right)$ represents the Fourier transform operation. $A\left(\cdot \right)$ and $P\left(\cdot \right)$ are amplitude and phase spectra functions, respectively, and ${e}^{-10}$ is a constant. Then, we obtain the logarithmic amplitude ${L}_{tgt}$ based on ${A}_{tgt}$. A mean filter with a kernel size of $3 \times 3$, a stride of 1, and a padding of 1 is used to obtain the mean spectrum $\stackrel{`}{{L}_{tgt}}$ from ${L}_{tgt}$. Subsequently, the residual spectrum ${R}_{tgt}$ is computed as the difference between ${L}_{tgt}$ and $\stackrel{`}{{L}_{tgt}}$, followed by a Fourier inverse transform combined with the phase spectrum ${P}_{tgt}$ to yield ${E}_{tgt}$. ${E}_{tgt}$ is then passed through a Gaussian smoothing filter to generate the saliency map (${S}_{tgt}$) as follows,

2 $${S}_{tgt}=G*\left({\text{IFFT}}\left({\left[\text{exp}\left({R}_{tgt}+{P}_{tgt}\right)\right]}^{2}\right)\right),$$

Where ${\text{IFFT}}\left(*\right)$ denotes the inverse Fourier transform and $G$ denotes the Gaussian smoothing filter. Finally, we merge the temporal domain features ${I}_{tgt}$ and the frequency domain features ${S}_{tgt}$ along the channel dimension and obtain the fused feature map ${O}_{tgt}$ through the convolution layer.

Once we obtain the fused feature map ${O}_{tgt}$, we partition the image into multiple patches of dimension $16\times 16\hspace{0.25em}{\text{pixels}}$ which are transformed into a one-dimensional embedding sequence via path embedding and position embedding. Then, we leverage multiple stacked Transformer layers, containing Multi-head Self-Attention (MSA) and Multi-Layer Perception (MLP) blocks, to extract the features $Z$ as follows,

3 $${Z}^{l}=MSA\left({Z}^{l-1}\right)+MLP\left(MSA\left({Z}^{l-1}\right)\right).$$

Additionally, layer normalization is implemented before the MSA and MLP blocks for brevity, although it is not explicitly depicted in the formula. This paper employs ${Z}^{1},{Z}^{2},\cdots ,{Z}^{L}$ as the features of the Transformer layer $l=1\dots Z$.

#### Multi-branch decoder

To foster interaction across features from different layers, we introduce a multi-branch aggregation design that progressively integrates corresponding contexts from different layers. In our model, we choose features from layers 2, 5, 8, and 11, named ${Z}^{2},{Z}^{5},{Z}^{8}$, and ${Z}^{11}$. Initially, we reshape the features from a 2D shape ($\frac{HW}{512}\times C$) to a 3D shape ($\frac{H}{16}\times \frac{W}{16}\times C$). Then, these features pass through three convolutional layers with kernel sizes of $1 \times 1$, $3 \times 3$, and $3 \times 3$, respectively. To promote interactions among branches, we introduce a top-down aggregation structure, merging top and bottom layer features via element-wise summation and expanding the size dimensions through an up-sampling operation. Once we obtain four sets of combined features, we concatenate them along the channel dimension and rescale their size to the input dimensions using convolutional and up-sampling operations. Based on multi-branch features, we generate the predicted organoid segmentation mask ${M}_{tgt}$, where the channel of P is 2.

#### Biological knowledge-driven branch

TransOrga-plus offers a knowledge-driven branch to interactively integrate feedback from biologists, as shown in Fig. 5. Biologists provide biological information through visual annotations $${I}_{bio}$$, such as single-organoid regions and medium regions. Given a target image input $${I}_{tgt}$$ and the initial biological information $${I}_{bio}$$. The multi-modal encoder extracts visual features $${E}_{tgt}$$ and $${E}_{bio}$$ from $${I}_{tgt}$$ and $${I}_{bio}$$, respectively. The biological-driven knowledge branch introduces the extracted biological knowledge $${E}_{bio}$$ into the model. $B$ contains the biological knowledge and the original bright-field image features. Finally, the decoder utilizes the combined features $$B$$ and outputs target recognition $${\varvec{M}}$$ and tracking $${\varvec{T}}$$ results. The mathematical formulation for this branch can be found as follows,

4 $$\begin{aligned}&{E}_{tgt}={\text{Encoder}}\left({I}_{tgt}\right)\\&{E}_{bio}={\text{Encoder}}\left({I}_{bio}\right)\\&B=\text{BioEncoder }\left({E}_{tgt},{E}_{bio}\right)\\&M,T={\text{Decoder}}\left(B\right).\\ \end {aligned}$$

#### Tracking module

To effectively track high-throughput organoids from microscopic image sequences, we utilize the decoupling features in the hierarchical propagation (DeAOT) method [40], which leverages hierarchical propagation with decoupled feature representation. Given a sequence of microscopic images and a reference annotation ${M}_{t}$, DeAOT propagates this annotation across the entire sequence, enabling consistent object tracking over time.

The core innovation of DeAOT lies in its decoupling of visual features and identification features into two distinct branches: the Visual Branch and the Identification Branch. In the Visual Branch, organoids are matched across frames via attention maps computed on patch-wise visual embeddings. These attention maps guide the propagation of visual features from previously stored frames to the current frame. Importantly, this propagation is entirely decoupled from identification embeddings, which ensures that the visual embeddings remain unbiased and purely appearance-based. The visual feature propagation is formulated as follows:

5 $$\begin{aligned}\widetilde{{I}_{l}^{t}}&={\text{Att}}\left({I}_{l}^{t}{W}_{l}^{K},{I}_{l}^{m}{W}_{l}^{K},{I}_{l}^{m}{W}_{l}^{V}\right)\\&={\text{Corr}}\left({I}_{l}^{t}{W}_{l}^{K},{I}_{l}^{m}{W}_{l}^{K}\right){I}_{l}^{m}{W}_{l}^{I},\end {aligned}$$

Where ${I}_{l}^{t}$ is the visual embedding of $l-th$ propagation layer at $t-th$ image and ${I}_{l}^{m}$ is the stored visual embeddings of $m$ images. ${W}_{l}^{K}$ and ${W}_{l}^{V}$ are parameters to project visual features into matching space and propagation space, respectively. The Identification Branch, in parallel, handles the propagation of object-specific identity features. It utilizes both the stored mask features and additional identification information to ensure that each organoid is consistently recognized across frames. This is formulated as:

6 $$\begin{array}{c}\widetilde{{M}_{l}^{t}}={\text{Att}}({I}_{l}^{t}{W}_{l}^{K},{I}_{l}^{m}{W}_{l}^{K},{M}_{l}^{m}{W}_{l}^{\overline{V} }+I D({Y}^{m}))\\={\text{Corr}}\left({I}_{l}^{t}{W}_{l}^{K},{I}_{l}^{m}{W}_{l}^{K}\right)\left({M}_{l}^{m}{W}_{l}^{\overline{V} }+ID\left({Y}^{m}\right)\right),\end{array}$$

Where ${M}_{l}^{m}$ and ${Y}^{m}$ is the stored identification features of $m$ masks. $ID\left(\cdot \right)$ is the identification method [40]. DeAOT enables accurate and real-time tracking of high-throughput organoids, preserving both spatial continuity and object identity across complex dynamic sequences.

#### Analysis module

After obtaining segmentation results, we can perform single-organoid analysis.

*How to obtain single-organoid:* Typically, neural network image segmentation methods apply an absolute threshold to predicted pixels to generate a binary detection mask. While effective, this approach ignores valuable prediction confidence information. During training, the network learns to produce a 2-pixel boundary from the training dataset. Consequently, the network predictions have slightly less confidence in pixels near organoid boundaries. First, we compute the partial derivative of pixel intensity to detect high-contrast regions. Second, we apply a blurring method to smooth noisy regions. Third, we employ a hysteresis-based threshold to identify locally strong edges. Subsequently, edges are removed from the thresholder prediction image to designate the centres of each organoid. These centres serve as the initial basins in a watershed transformation to segment contacting organoids. The image undergoes further refinement to eliminate organoids potentially outside the field-of-view or below a specific size threshold. The pipeline yields a labelled image wherein pixels representing individual organoids are assigned a unique organoid identifier (ID) number, facilitating subsequent single-organoid analysis.

For single-organoid analysis, the grouping of pixels in the segmentation mask is essential to identify individual organoids. For isolated organoids, this task is straightforward since all high-confidence pixels in a cluster correspond to one organoid. However, for organoids that are in physical contact, it becomes more challenging. To address this issue, we introduce an organoid separation pipeline that leverages the raw network prediction to group pixels into single-organoid clusters.

For time-course analysis, we measure changes such as the size and shape of individual organoids over time. Based on the single-organoid analysis for each frame, our tracking module consistently follows the individual organoids between different frames and outputs time-course organoid analysis.

#### Losses

As the first step in the system, the output quality of segmentation influences the down-stream processing. Considering the characteristics of organoids, we design a series of loss functions, including the focal loss ${\mathcal{L}}_{focal}$, the dice loss ${\mathcal{L}}_{dice}$ and the compact loss ${\mathcal{L}}_{com}$ as the weighted loss function in segmentation optimization objective. Due to the imbalanced ratio between organoids and background in the image, we utilize the focal loss ${\mathcal{L}}_{focal}$ to penalize the error-predicted mask at pixel level as follows,

7 $$\begin{aligned}& {\mathcal{L}}{focal}=-{\alpha }{\left(1-{pt}\right)}^{\upgamma }\text{log}\left({pt}\right)\\ & {\text{p}}{t}=\left\{\begin{array}{c}\widehat{\text{p}} \, {\text{if}} \, argmax\left({M}_{\text{tgt}}\right)=1\\ 1-\widehat{\text{p}} \, {\text{otherwise,}}\end{array}\right.\end {aligned}$$

Where $argmax\left(\cdot \right)$ obtains the channel index which has the maximum value. $\widehat{p}$ is the predicted probability of the organoid class. ${\alpha }$ and $\upgamma$ are hyperparameters for the sample weight and the weight for hard cases, respectively. The dice loss ${\mathcal{L}}_{dice}$ measures the ratio of the overlap between the predicted mask and ground-truth mask to their union as follows,

8 $${\mathcal{L}}_{\text{dice }}=1-\frac{2\left|\textrm{argmax}\left({M}_{\textrm{tgt}}\right)\cap {G}_{\text{tgt}}\right|}{\left|\left({M}_{\text{tgt}}\right)\right|\cup \left|{G}_{\text{tgt}}\right|},$$

Where ${G}_{tgt}$ is the ground-truth segmentation mask. Since most organoids are circles or ellipses, which are highly compact. We take advantage of this phenomenon and introduce compact loss ${\mathcal{L}}_{com}$ as follows,

9 $${\mathcal{L}}_{com}=\frac{\sum_{\text{i}\epsilon\Omega } \sqrt{{\left({\nabla }_{\text{pih}}\right)}^{2}+{\left({\nabla }_{\text{p}}\right)}^{2}}+\varepsilon }{4\pi \left(\sum_{\text{i}\in\Omega } \left|{\text{p}}_{\text{i}}\right|\right)+\varepsilon }$$

Where ${p}_{i}$ is the predicted segmentation results, $\Omega$ is the pixel set of predicted segmentation mask. $\nabla {p}_{ih}$ and $\nabla {p}_{iv}$ are the gradients for pixels in the horizontal and vertical directions. $\upepsilon$ is a hyperparameter. Above all, we combine all these losses to form the final loss as follows,

10 $${\mathcal{L}}_{\text{final }}={\lambda }_{1}{\mathcal{L}}_{\text{focal }}+{\lambda }_{2}{\mathcal{L}}_{\text{dice }}+{\lambda }_{3}{\mathcal{L}}_{\text{com}}$$

#### Implementation details

The proposed model is implemented using Python 3.9 and PyTorch 1.13 and trained on Ubuntu 20.04 with NVIDIA GeForce RTX 3090 for 100 epochs. We train our model using the stochastic gradient descent algorithm with a stochastic weight-averaging strategy. Dropout is enabled during the training and testing for uncertainty estimation. The initial learning rate is 0.01, and we reduce it by a factor 0.1 every 10 epochs. Our dataset is randomly split into training, validation and test sets (70%, 20%, 10%, respectively). The validation set is used to improve our models and select the best model hyperparameters. In our experiments, we set ${\lambda }_{1}$ as 1.0, ${\lambda }_{2}$ as 0.5 and ${\lambda }_{3}$ as 0.5. The detailed guidelines about how to train our model on private dataset is shown in https://github.com/dev-csftan/TransOrga.

## Supplementary Information

Additional file 1: Fig. S1. Comparison results on unseen high-resolution bright-field microscopic organoids images. Fig. S2. Comparison Results of organoid tracking using the microscopic image sequence.

## Data Availability

The access of the organoid data analysed in this manuscript is provided by the Merck Group. The data, code and demo for this paper are available at [https://zenodo.org/records/16900123] (https://zenodo.org/records/16900123) [40].

## Abbreviations

TU/e: Eindhoven University of Technology
ACC: Salivary adenoid cystic carcinoma
Colon: Colon epithelia
Lung: Lung epithelia
PDAC: Pancreatic ductal adenocarcinoma
Mammary: Mammary
mIoU: Mean intersection over union
SOTA: State-of-the-art
STA: Segment-and-track anything
SAM: Segment anything model
MSA: Multi-head self-attention
MLP: Multi-layer perception
DeAOT: Decoupling features in the hierarchical propagation
ID: Identifier
