# Supplementary material for: A knowledge-driven deep learning framework for organoid morphological segmentation and characterization
Source: BMC Biol. 2025 Oct 21;23:313. doi: 10.1186/s12915-025-02411-8 (PMC12538981; doi:10.1186/s12915-025-02411-8)
Supplement: Supplementary file 1 — Additional file 1: Fig. S1. Comparison results on unseen high-resolution bright-field microscopic organoids images. Fig. S2. Comparison Results of organoid tracking using the microscopic image sequence. [file 12915_2025_2411_MOESM1_ESM.docx]

**ADDITIONAL FILE 1.**

**TABLE OF CONTENTS**

pg. nos.

**Additional Figures**

Figure S1. Comparison results on unseen high-resolution bright-field microscopic organoids images. 2

Figure S2. Comparison Results of organoid tracking using the microscopic image sequence. 3


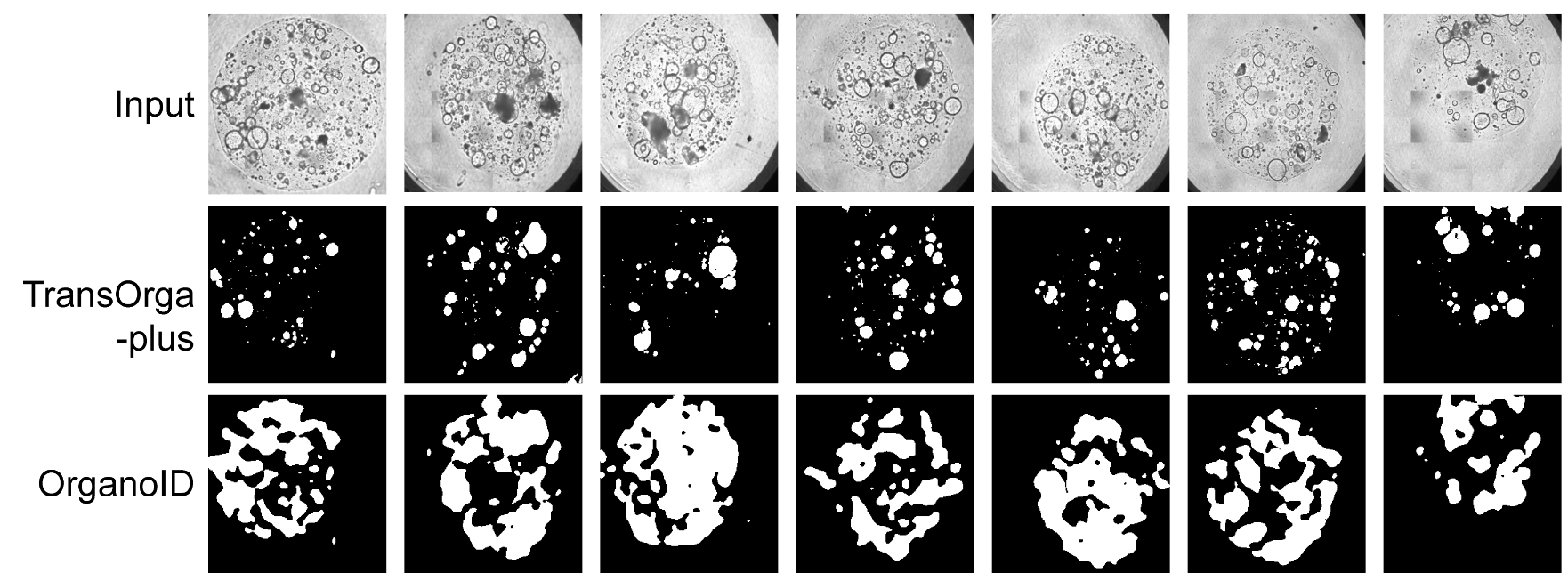


**Figure S1. Comparison results on unseen high-resolution bright-field microscopic organoids images.** The results indicate that our method maintains high robustness even with unseen ultra-high-resolution images.

**
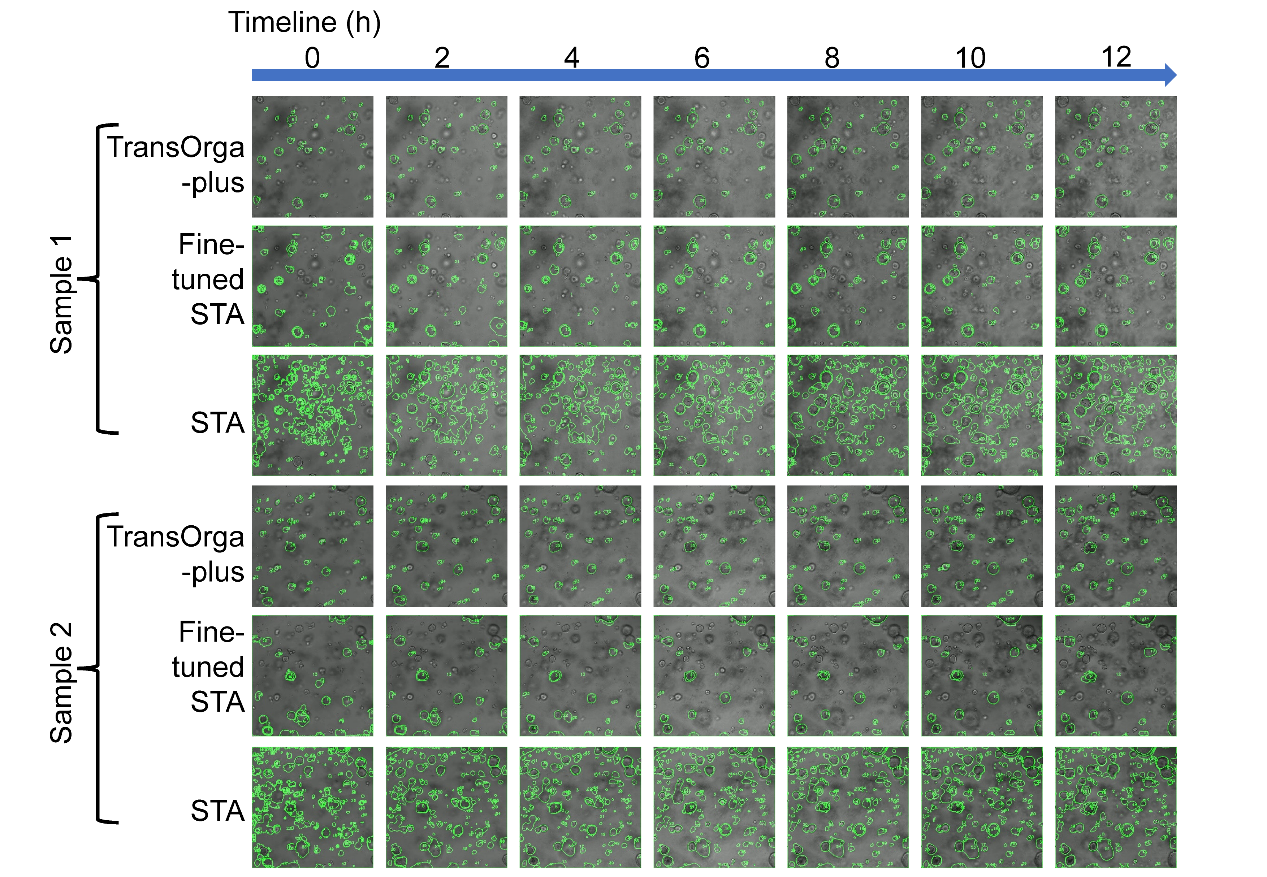
**

**Figure S2. Comparison Results of organoid tracking using the microscopic image sequence.** We compare ours with original STA (Segment and Tracking Anything) and the fine-tuned STA. We use green circles to indicate organoid cells. The results indicate that our method achieves better tracking performance.
